# Bloqueadores do Receptor de Angiotensina Avaliados por Medida de Consultório e Residencial da Pressão Arterial. Estudo TeleMRPA

**DOI:** 10.36660/abc.20210504

**Published:** 2022-04-26

**Authors:** Weimar Kunz Sebba Barroso, Andréa Araujo Brandão, Priscila Valverde de Oliveira Vitorino, Audes Diógenes de Magalhães Feitosa, Eduardo Costa Duarte Barbosa, Roberto Dischinger Miranda, Josep Redon, Miguel Camafort-Babkowski, Antonio Coca, Marco Antônio Mota Gomes

**Affiliations:** 1 Universidade Federal de Goiás Liga de Hipertensão Arterial Goiânia GO Brasil Universidade Federal de Goiás - Liga de Hipertensão Arterial, Goiânia, GO – Brasil; 2 Universidade do Estado do Rio de Janeiro Rio de Janeiro RJ Brasil Universidade do Estado do Rio de Janeiro – Cardiologia, Rio de Janeiro, RJ – Brasil; 3 Pontifícia Universidade Católica de Goiás Escola de Ciências Sociais e da Saúde Goiânia GO Brasil Pontifícia Universidade Católica de Goiás - Escola de Ciências Sociais e da Saúde,Goiânia, GO – Brasil; 4 Universidade de Pernambuco Recife PE Brasil Universidade de Pernambuco, Recife, PE – Brasil; 5 Universidade Católica de Pernambuco Recife PE Brasil Universidade Católica de Pernambuco, Recife, PE – Brasil; 6 Complexo Hospitalar Santa Casa de Misericórdia de Porto Alegre Porto Alegre RS Brasil Complexo Hospitalar Santa Casa de Misericórdia de Porto Alegre – Cardiologia, Porto Alegre, RS - Brasil; 7 Universidade Federal de São Paulo Escola Paulista de Medicina São Paulo SP Brasil Universidade Federal de São Paulo - Escola Paulista de Medicina, São Paulo, SP – Brasil; 8 University of Valencia Valencia Espanha University of Valencia – Hypertension, Valencia, Comunitat Valenciana – Espanha; 9 University of Barcelona Hospital Clínic Hypertension Unit Barcelona Catalunya Espanha University of Barcelona - Hospital Clínic. Hypertension Unit, Barcelona, Catalunya – Espanha; 10 Hypertension and Vascular Risk Unit Hospital Clinic University of Barcelona Barcelona Espanha Hypertension and Vascular Risk Unit. Hospital Clinic. University of Barcelona,Barcelona – Espanha; 11 Centro Universitário CESMAC Hospital do Coração Maceió AL Brasil Centro Universitário CESMAC - Hospital do Coração, Maceió, AL – Brasil

**Keywords:** Hipertensão, Bloqueadores do Receptor Tipo 1 de Angiotensina II, Losartana, Anti-Hipertensivos/uso terapêutico, Idade, Sexo, Peso e Medidas

## Abstract

**Fundamento:**

O tratamento adequado e a obtenção das metas na hipertensão arterial são importantes na redução dos desfechos cardiovasculares.

**Objetivos:**

Descrever os bloqueadores do receptor de angiotensina (BRA) em monoterapia ou combinação dupla e a taxa de controle da hipertensão arterial.

**Métodos:**

Estudo transversal que avaliou pacientes em uso de BRA entre 2017 e 2020. Foram excluídos aqueles em uso de três ou mais anti-hipertensivos. As variáveis analisadas foram: sexo, idade, índice de massa corporal, medidas válidas da medida residencial da pressão arterial (MRPA); pressão arterial sistólica (PAS) e diastólica (PAD) obtidas pela MRPA e de forma casual; variabilidade pressórica; classe dos anti-hipertensivos e dos BRAs. Foram utilizados testes de *t* pareado, qui-quadrado e Fisher, além de sobreposição dos intervalos de confiança de 95% com nível de significância de 5% (p < 0,05).

**Resultados:**

Foram selecionados 17.013 pacientes; destes, 12.813 preencheram os critérios, dos quais 62,1% eram do sexo feminino. O número médio de medidas válidas foi de 23,3 (±2,0), com médias para a PAS de 126,8±15,8 mmHg e 133,5±20,1 mmHg (p < 0,001) e para a PAD de 79,1±9,7 mmHg e 83,6±11,9 mmHg (p < 0,001) pela MRPA e medida casual, respectivamente. Losartana foi o BRA mais utilizado e o que apresentou comportamentos mais elevados da pressão arterial. As combinações de BRA com diuréticos ou com antagonistas de canal de cálcio tiveram menores valores de pressão arterial.

**Conclusões:**

Losartana foi utilizada em mais da metade dos pacientes, apesar de ser a menos eficiente na redução e no controle da pressão arterial.

## Introdução

O tratamento e controle adequado da hipertensão arterial (HA) ainda hoje é um dos grandes desafios no tratamento dessa doença, que é a principal causa de morte em todo o mundo. A adoção de estratégias de tratamento alinhadas com as evidências científicas mais atuais é um dos caminhos para otimizar esses resultados.^1 - 3^ Nesse contexto, o uso de fármacos com características capazes de atuar de forma efetiva na redução da pressão arterial (PA), com consequente proteção quanto aos principais desfechos relacionados à doença hipertensiva, e ainda ter a capacidade de permitir uma única tomada ao dia, em decorrência de uma meia-vida longa, sem interferência negativa nos parâmetros metabólicos, é o que se espera para a obtenção dos melhores resultados com o tratamento instituído. Além disso, sabe-se que pequenas reduções da PA, mesmo nas fases iniciais da HA, são capazes de promover redução nos principais desfechos cardiovasculares.^1 , 4 , 5^

Por outro lado, apesar de todas essas evidências, encontramos na cesta básica de medicamentos ofertados pelo Sistema Único de Saúde (SUS) fármacos de meia-vida curta, em monoterapia e com a necessidade de várias tomadas ao dia; características que podem impactar negativamente na adesão e dificultar o controle adequado da PA. Destaca-se que a realidade do SUS reflete o contexto das estratégias medicamentosas adotadas em nosso país para 75% dos pacientes hipertensos.^1 , 6^

Artigo publicado em 2021 que avaliou uma base de dados de 22.446 indivíduos submetidos a medida da PA no consultório e no domicílio, dos quais 11.337 eram hipertensos tratados por cardiologistas com fármacos anti-hipertensivos, identificou que, em 74,6% dos casos, o bloqueio do sistema renina-angiotensina-aldosterona foi a estratégia adotada, sendo que o uso dos bloqueadores dos receptores de angiotensina (BRAs) ocorreu em 58,7%, seja em monoterapia ou em combinação.^7^

Foram objetivos do presente estudo: (i) verificar a distribuição da prescrição dos BRAs em monoterapia e combinação total e por sexo, região geográfica e presença de diabetes; (ii) comparar a taxa de controle da PA segundo a medida casual e a medida residencial da PA (MRPA) para todas as estratégias de tratamento medicamentoso com BRA; (iii) comparar o controle da PA entre a MRPA e a medida casual; e (iv) comparar as médias de pressão arterial sistólica (PAS), pressão arterial diastólica (PAD), pressão de pulso (PP) e variabilidade da PA obtidas com utilização de BRAs em monoterapia ou em combinação dupla, considerando a classe como um todo e os vários tipos de medicamentos que a compõem.

## Métodos

Este estudo foi aprovado pelo Comitê de Ética em Pesquisa Humana do Hospital das Clínicas da Universidade Federal de Goiás sob CAE número 99691018.7.0000.5078 e avaliou pacientes que realizaram exames na plataforma TeleMRPA (www.telemrpa.com) de maio de 2017 até outubro de 2020.

A plataforma foi desenvolvida como ferramenta de laudo a distância por telemonitoramento, com características que permitem a análise e o filtro do banco de dados de acordo com as perguntas científicas que se pretende investigar. O algoritmo matemático utilizado possibilita a análise com proteção dos dados pessoais do paciente, assim como das clínicas ou unidades de saúde, seja para a interpretação do exame, seja para a construção de projetos de pesquisa. Por não se tratar de um *software* e sim de uma plataforma acessível em qualquer terminal de computador, *tablet* ou *smartphone* , a inserção dos dados relativos à medida da PA pode ser feita de forma remota e simplificada.^8^

**Figura 1:**
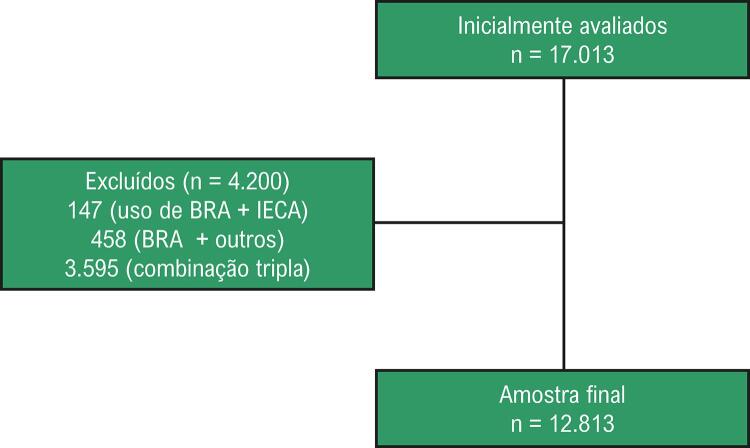
– *Fluxograma de seleção da amostra do estudo. BRA: bloqueadores dos receptores de angiotensina; IECA: inibidores da enzima conversora de angiotensina.*

A base de dados analisada se restringiu aos pacientes que estavam em uso de BRAs. Foram incluídos pacientes com idade igual ou superior a 18 anos e em uso de monoterapia ou combinação dupla. Foram excluídos pacientes em uso de combinação de três ou mais anti-hipertensivos ou em combinação com inibidores da enzima conversora de angiotensina ou combinados com anti-hipertensivos de uso pouco frequente nas combinações duplas (espironolactona, vasodilatadores diretos, alfa2 agonistas) ( Figura 1 ). Em relação aos BRAs, optamos por excluir da apresentação dos resultados a irbesartana, por representar um n muito pequeno em relação à amostra final.

Foram utilizados os seguintes dados da plataforma TeleMRPA: sexo (masculino/feminino); idade (em anos, calculada a partir da data de nascimento); índice de massa corporal (IMC); número de medidas válidas da MRPA; PAS e PAD obtidas pela MRPA e de forma casual; variabilidade pressórica pela MRPA obtida a partir do desvio padrão das 24 medidas domiciliares durante o protocolo; classe dos medicamentos utilizados; e os fármacos da classe dos BRAs.

Também foi avaliada a distribuição da amostra por regiões geográficas do Brasil, assim como a prevalência de indivíduos em uso de medicamentos para o tratamento de diabetes melito (antidiabéticos orais e/ou insulina).

Para o cálculo do IMC, foram utilizados o peso e a altura aferidos e a fórmula de Quetelet.^9^ Para a medida da MRPA, o aparelho foi disponibilizado para o paciente, que foi orientado sobre o manuseio e a técnica adequados para a medida da PA no dia da entrega do aparelho.^1^ Ainda nesse primeiro dia, foram realizadas duas medidas no ambiente da clínica/consultório e, nos 4 dias subsequentes, o paciente (e/ou cuidador/acompanhante) realizou as medidas em seu domicílio, conforme o protocolo. Considerou-se como medida casual a média das duas medidas do primeiro dia, e como medida domiciliar a média das 24 medidas do segundo ao quinto dia.^8 , 10^

Foram utilizados aparelhos automáticos validados das marcas Omron, Geratherm e Microlife.

Os dados foram exportados da plataforma TeleMRPA para o Excel. Todas as classes de medicamentos descritas na plataforma foram revisadas e codificadas por duas equipes de trabalho; em seguida, os bancos de dados foram cruzados para identificação de dados discrepantes, que, quando presentes, foram revisados com toda a equipe e a coordenação. Foram considerados com PA controlada os indivíduos com a PA casual menor que 140 mmHg e 90 mmHg e, pela MRPA, com valores menores que 130 mmHg e 80 mmHg para a PAS e PAD, respectivamente.^1^

### Análise estatística

A análise estatística foi realizada utilizando o *software* Stata, versão 14.0. As variáveis quantitativas foram apresentadas com média e desvio padrão, e as qualitativas com frequência absoluta e relativa. Para a verificação da normalidade dos dados, utilizou-se o teste de Kolmogorov-Smirnov.

Nas comparações das médias de PAS, PAD e PP obtidas na MRPA e na medição casual, utilizou-se o teste t de Student pareado, e os testes qui-quadrado ou de Fisher foram utilizados para comparar as taxas de controle obtidas por meio da medição casual com aquelas identificadas pela MRPA, e também para comparar as taxas de controle e de não controle da PA segundo a utilização ou não de cada estratégia medicamentosa, considerando a medida casual e a MRPA.

A sobreposição dos intervalos de confiança de 95% foi utilizada para comparar as diferenças entre as médias de PAS, PAD, PP e a variabilidade da PA obtidas com a utilização de BRAs em monoterapia ou em combinação dupla, considerando a classe como um todo e os vários tipos de medicamentos que a compõem. Foi adotado um nível de significância de 5% (p < 0,05).

## Resultados

Foram avaliados 12.813 pacientes, a maioria do sexo feminino, e quase a metade da região Nordeste. A prevalência de diabetes foi de 6,2% ( Tabela 1 ).

**Tabela 1:** – Descrição dos pacientes hipertensos em uso de BRA (classe), n = 12.813

Variável	Total n (%)	BRA n (%)	BRA + DIU n (%)	BRA + BB n (%)	BRA + ACC n (%)
	**12.813 (100)**	**6.225 (48,6)**	**3.006 (23,5)**	**1.433 (11,2)**	**2.149 (16,8)**
**Sexo**					
Feminino	7.953 (62,1)	3.749 (60,2)	2.006 (66,7)	980 (68,4)	1.218 (56,7)
Masculino	4.860 (37,9)	2.476 (39,8)	1.000 (33,2)	453 (31,6)	931 (43,3)
**Região**					
Não identificada	37 (0,3)	12 (0,2)	16 (0,5)	05 (0,3)	04 (0,1)
Nordeste	6.347 (49,6)	3.187 (51,2)	1.355 (45,1)	698 (48,7)	1.107 (51,5)
Norte	802 (6,3)	326 (5,2)	194 (6,5)	52 (3,6)	230 (10,7)
Centro-Oeste	1.003 (7,8)	478 (7,7)	232 (7,7)	162 (11,3)	131 (6,1)
Sudeste	4.028 (31,4)	1.961 (31,5)	1.026 (34,1)	444 (31,0)	597 (27,8)
Sul	596 (4,7)	261(4,2)	183 (6,1)	72 (5,0)	80 (3,7)
**Diabetes**					
Não	12.015 (93,8)	5.877 (94,4)	2.811 (93,5)	1.294 (90,3)	2.033 (94,6)
Sim	798 (6,2)	348 (5,6)	195 (6,5)	139 (9,7)	116 (5,4)

*ACC: antagonistas dos canais de cálcio; BB: betabloqueadores; BRA: bloqueadores dos receptores de angiotensina; DIU: diuréticos.*

A estratégia de tratamento encontrada na amostra se dividiu em 48,5% dos pacientes em uso de monoterapia e 51,2% com combinações duplas. Os fármacos que compuseram a classe dos BRAs apresentaram a seguinte distribuição: 57,2%, losartana; 18,8%, olmesartana; 15,0%, valsartana; 4,8%, telmisartana; 3,8%, candesartana; e 0,4%, irbesartana.

O número médio de medidas válidas da MRPA foi de 23,3 (±2,0). As diferenças nos valores médios entre PA casual e MRPA para a PAS e PAD foram de 6,7 mmHg (p < 0,001) e 4,5 mmHg (p < 0,001), respectivamente. Essas diferenças caracterizam o efeito do avental branco e se mantêm em todas as estratégias de tratamento. Esse comportamento se repete com todos os BRAs, seja em monoterapia ou combinação. Também avaliamos e comparamos o percentual de controle pela medida casual e pela MRPA em monoterapia e por estratégia de combinação ( Tabela 2 ).

**Tabela 2:** – Descrição da amostra e comparação do controle da pressão arterial pela medida casual e pela MRPA segundo a utilização de BRA em monoterapia e combinações, n = 12.813

Variável	MRPA	Casual	p*
**Total (n = 12.813)**			
PAS	126,8±15,8	133,5±20,1	< 0,001
PAD	79,1±9,7	83,6±11,9	< 0,001
PP	52,2±14,4	49,9±16,1	< 0,001
**BRA (monoterapia) (n = 6.225)**			
PAS	126,9±15,6	133,5±19,8	< 0,001
PAD	79,7±9,6	84,3±11,7	< 0,001
PP	51,7±14,0	49,2±15,7	< 0,001
**BRA + DIU (n = 3.006)**			
PAS	125,0±15,8	132,3±20,3	< 0,001
PAD	78,6±9,5	83,3±11,9	< 0,001
PP	50,7±14,3	49,1±16,1	< 0,001
**BRA + ACC (n = 2.149)**			
PAS	127,0±14,9	133,8±19,2	< 0,001
PAD	78,4±9,9	82,8±11,9	< 0,001
PP	53,2±14,0	51,0±15,8	< 0,001
**BRA + BB (n = 1.433)**			
PAS	129,4±17,9	136,0±22,2	< 0,001
PAD	78,3±10,4	82,6±12,4	< 0,001
PP	56,0±16,2	53,4±17,7	< 0,001
**Variável**	**Controlado**	**Não controlado**	p**
**Total**			
MRPA	5.695 (44,5)	7.118 (55,5)	< 0,001
Medida casual	7.211 (56,3)	5.602 (43,7)	
**BRA monoterapia**			
MRPA	2.691 (43,2)	3.534 (56,8)	0,007
Medida casual	3.485 (56,0)	2.740 (44,0)	0,513
**BRA + DIU**			
MRPA	1.441 (48,0)	1.565 (52,1)	< 0,001
Medida casual	1.751 (58,3)	1.255 (41,7)	0,013
**BRA + ACC**			
MRPA	960 (44,7)	1.189 (55,3)	0,818
Medida casual	1.204 (56,0)	945 (44,0)	0,796
**BRA + BB**			
MRPA	603 (42,1)	830 (57,9)	0,056
Medida casual	771 (53,8)	662 (46,2)	0,045

*Teste t pareado; **teste qui-quadrado ou exato de Fisher. ACC: antagonistas dos canais de cálcio; BB: betabloqueadores; BRA: bloqueadores dos receptores de angiotensina; DIU: diuréticos; MRPA: medida residencial da pressão arterial; PAD: pressão arterial diastólica; PAS: pressão arterial sistólica; PP: pressão de pulso.

Na Tabela 3 está descrito o valor médio da PA pela medida casual e pela MRPA, assim como o percentual de controle com os diferentes BRAs em monoterapia e, nas Tabelas 4 , 5 e 6 , com as combinações de BRAs com diuréticos (DIU), antagonistas dos canais de cálcio (ACC) e betabloqueadores (BBs), respectivamente.

**Tabela 3:** – Descrição da amostra e comparação do controle da pressão arterial pela medida casual e pela MRPA segundo utilização dos tipos de BRA em monoterapia, n = 6.225

Variável	MRPA	Medida casual	p*
**Losartana (n = 3.861)**			
PAS	128,3 ±15,8	135,4± 20,3	< 0,001
PAD	80,6±9,7	85,5±11,8	< 0,001
PP	52,1±14,1	50,0±16,0	< 0,001
**Valsartana (n = 818)**			
PAS	126,8±15,3	132,4±19,5	< 0,001
PAD	78,6±9,5	82,4±10,8	< 0,001
PP	52,7±14,3	50,0±16,0	< 0,001
**Candesartana (n = 221)**			
PAS	124,0±12,9	129,0±17,0	< 0,001
PAD	77,5±7,8	81,4±9,5	< 0,001
PP	50,9±13,4	47,6±14,8	< 0,001
**Olmesartana (n = 1.032)**			
PAS	123,0±14,9	128,4±18,1	< 0,001
PAD	77,9±9,4	82,0±11,9	< 0,001
PP	49,8±13,0	46,4±14,1	< 0,001
**Telmisartana (n = 287)**			
PAS	126,2±14,8	132,6±18,0	< 0,001
PAD	79,6±9,1	84,0±11,3	< 0,001
PP	51,1±13,9	48,3±15,1	< 0,001
**Variável**	**Controlado**	**Não controlado**	p**
**Losartana**			
MRPA	1.517 (39,3)	2.344 (60,7)	< 0,001
Casual	1.984 (51,4)	1.877 (48,6)	< 0,001
**Valsartana**			
MRPA	369 (45,1)	449 (54,9)	0,693
Casual	489 (59,8)	329 (40,2)	0,037
**Candesartana**			
MRPA	111 (50,2)	110 (49,8)	0,081
Casual	150 (67,9)	71 (32,1)	< 0,001
**Olmesartana**			
MRPA	559 (54,2)	473 (45,8)	< 0,001
Casual	682 (66,1)	350 (33,9)	< 0,001
**Telmisartana**			
MRPA	130 (45,3)	157 (54,7)	0,770
Casual	172 (59,9)	115 (40,1)	0,207

*Teste t pareado; **teste qui-quadrado ou exato de Fisher. BRA: bloqueadores dos receptores de angiotensina; MRPA: medida residencial da pressão arterial; PAD: pressão arterial diastólica; PAS: pressão arterial sistólica; PP: pressão de pulso.

**Tabela 4:** – Descrição da amostra e comparação do controle da pressão arterial pela medida casual e pela MRPA segundo utilização dos tipos de BRA em combinações duplas com DIUs, n = 3.006

Variável	MRPA	Medida casual	p
**Olmesartana + DIU (n = 530)**			
PAS	122,1±15,8	128,4±20,2	< 0,001
PAD	77,0±9,6	81,1±12,0	< 0,001
PP	49,5±15,1	47,3±16,3	< 0,001
**Candesartana + DIU (n = 151)**			
PAS	123,1±5,0	130,9±20,8	< 0,001
PAD	77,6±9,1	82,4±12,1	< 0,001
PP	49,6±14,1	48,5±15,1	0,199
**Telmisartana + DIU (n = 123)**			
PAS	124,9±16,7	132,5±20,1	< 0,001
PAD	78,3±8,5	83,6±11,1	< 0,001
PP	51,1±15,9	48,9±16,8	< 0,001
**Valsartana + DIU (n = 1.920)**			
PAS	126,9±15,5	132,7±20,1	< 0,001
PAD	78,3±9,7	82,1±11,7	< 0,001
PP	53,2±14,3	50,6±16,1	< 0,001
**Losartana + DIU (n = 1.715)**			
PAS	125,7±15,7	133,8±20,1	< 0,001
PAD	79,2±9,4	84,2±11,7	< 0,001
PP	50,9±14,1	49,6±16,1	< 0,001
Variável	Controlado	Não controlado	p**
**Olmesartana + DIU**			
MRPA	288 (54,3)	242 (45,7)	< 0,001
Casual	335 (63,2)	195 (36,8)	0,001
**Candesartana + DIU**			
MRPA	80 (53,0)	71 (47,0)	0,034
Casual	99 (65,6)	52 (34,4)	0,021
**Telmisartana + DIU**			
MRPA	59 (48,0)	64 (52,0)	0,430
Casual	73 (59,4)	50 (40,6)	0,490
**Valsartana + DIU**			
MRPA	887 (46,2)	1.033 (53,8)	0,094
Casual	1.136 (59,2)	784 (40,8)	0,006
**Losartana + DIU**			
MRPA	779 (45,4)	936 (54,6)	0,382
Casual	965 (56,3)	750 (43,7)	0,992

*Teste t pareado; **teste qui-quadrado ou exato de Fisher. BRA: bloqueadores dos receptores de angiotensina; DIU: diuréticos; MRPA: medida residencial da pressão arterial; PAD: pressão arterial diastólica; PAS: pressão arterial sistólica; PP: pressão de pulso.

**Tabela 5:** – Descrição da amostra e comparação do controle da pressão arterial pela medida casual e pela MRPA segundo utilização dos tipos de BRA em combinações duplas com ACCs, n = 2.149

Variável	MRPA	Medida casual	p*
**Olmesartana + ACC (n = 626)**			
PAS	125,0±14,9	131,7±19,4	< 0,001
PAD	77,8±10,2	81,8±12,5	< 0,001
PP	51,9±14,5	49,9±15,9	< 0,001
**Candesartana + ACC (n = 419)**			
PAS	127,4±14,6	135,1±18,4	< 0,001
PAD	78,6±10,2	83,6±11,6	< 0,001
PP	53,4±13,9	51,5±15,4	< 0,001
**Telmisartana + ACC (n = 136)**			
PAS	128,7±15,8	132,4±18,8	0,003
PAD	78,6±10,3	81,8±11,7	< 0,001
PP	55,1±13,6	50,7±14,1	< 0,001
**Valsartana + ACC (n = 433)**			
PAS	127,0±15,2	132,6±19,5	< 0,001
PAD	77,4±9,6	80,7±11,6	< 0,001
PP	54,2±13,6	51,8±15,4	< 0,001
**Losartana + ACC (n = 903)**			
PAS	128,2±14,5	135,9±18,7	< 0,001
PAD	79,6±9,6	84,7±11,3	< 0,001
PP	53,1±3,7	51,1±15,9	< 0,001
**Variável**	**Controlado**	**Não controlado**	p**
**Olmesartana + ACC**			
MRPA	302 (48,2)	324 (51,8)	0,050
Casual	378 (60,4)	248 (39,6)	0,034
**Candesartana + ACC**			
MRPA	173 (41,3)	246 (58,7)	0,186
Casual	218 (52,0)	201 (48,0)	0,075
**Telmisartana + ACC**			
MRPA	69 (50,7)	67 (49,3)	0,138
Casual	84 (61,8)	52 (38,2)	0,195
**Valsartana + ACC**			
Casual	270 (62,4)	163 (37,6)	0,010
MRPA	206 (47,6)	227 (52,4)	0,183
**Losartana + ACC**			
MRPA	361 (40,0)	542 (60,0)	0,005
Casual	451 (49,9)	452 (50,1)	< 0,001

*Teste t pareado; **teste qui-quadrado ou exato de Fisher. ACC: antagonistas dos canais de cálcio; BRA: bloqueadores dos receptores de angiotensina; MRPA: medida residencial da pressão arterial; PAD: pressão arterial diastólica; PAS: pressão arterial sistólica; PP: pressão de pulso.

**Tabela 6:** – Descrição da amostra e comparação do controle da pressão arterial pela medida casual e pela MRPA segundo utilização dos tipos de BRA em combinações duplas com BBs, n = 1.433

Variável	MRPA	Medida casual	p*
**Olmesartana + BB (n = 230)**			
PAS	126,3±17,0	132,0±20,6	< 0,001
PAD	77,6±10,4	80,9±11,5	< 0,001
PP	53,6±14,8	51,1±17,0	< 0,001
**Candesartana + BB (n = 65)**			
PAS	129,8±17,3	133,8±21,0	< 0,001
PAD	75,8±11,8	79,1±14,2	0,012
PP	59,0±17,1	54,7±16,6	0,002
**Telmisartana + BB (n = 75)**			
PAS	128,4±16,5	132,6±21,9	0,01
PAD	78,0±10,7	82,0±13,9	< 0,001
PP	55,2±15,3	50,6±16,3	< 0,001
**Valsartana + BB (n = 213)**			
PAS	130,0±16,8	137,0±21,9	< 0,001
PAD	77,9±10,3	82,5±12,5	< 0,001
PP	57,0±15,7	54,5±18,0	< 0,001
**Losartana + BB (n = 851)**			
PAS	130,2±18,5	137,3±22,7	< 0,001
PAD	78,8±10,3	83,4±12,3	< 0,001
PP	56,2±16,7	53,8±17,9	< 0,001
Variável	Controlado	Não controlado	p**
**Olmesartana + BB**			
MRPA	114 (49,6)	116 (50,4)	0,115
Casual	138 (60,0)	92 (40,0)	0,251
Candesartana + BB			
MRPA	31 (47,7)	34 (52,3)	0,598
Casual	40 (61,5)	25 (38,5)	0,391
**Telmisartana + BB**			
MRPA	36 (48,0)	39 (52,0)	0,535
Casual	46 (61,3)	29 (38,7)	0,376
**Valsartana + BB**			
MRPA	91 (42,7)	122 (57,3)	0,610
Casual	113 (53,1)	100 (46,9)	0,338
**Losartana + BB**			
MRPA	331 (38,9)	520 (61,1)	0,001
Casual	433 (50,9)	418 (49,1)	0,001

*Teste t pareado; **teste qui-quadrado ou exato de Fisher. BB: betabloqueadores; BRA: bloqueadores dos receptores de angiotensina; MRPA: medida residencial da pressão arterial; PAD: pressão arterial diastólica; PAS: pressão arterial sistólica; PP: pressão de pulso.

Quando avaliamos o controle da PA de acordo com as metas menores que 140 mmHg e 90 mmHg para a medida casual e 130 mmHg e 80 mmHg para a MRPA, conforme diretrizes vigentes,^1^ a taxa de controle da PA na amostra total foi melhor quando obtida pela medida casual.

O controle da PA, considerando a MRPA, foi menor naqueles que utilizavam BRA em monoterapia e BRA com BB. Quando analisamos os tipos de BRA utilizados em monoterapia ou em combinação, o controle da PA foi menor considerando a MRPA naqueles que utilizavam losartana e maior nos que estavam em uso de BRA de meia-vida longa. Essa mesma análise para a medida casual em relação ao controle da PA repete essa tendência.

As taxas de controle dos diversos tipos de BRA combinados com ACC, BB ou DIU, tanto pela MRPA quanto pela medida casual, foram menores nas combinações com a losartana e maiores com BRAs de meia-vida longa.

**Figura 2:**
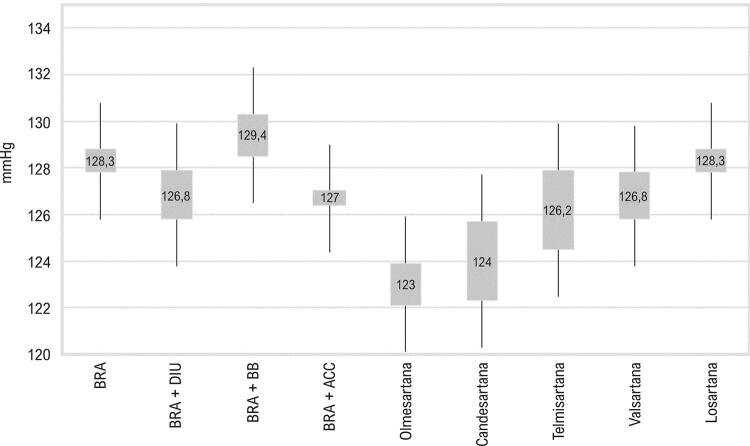
– *Comparação das médias de PAS (MRPA) obtidas com utilização de BRA (classes e tipos) em monoterapia ou em combinação dupla. ACC: antagonistas dos canais de cálcio; BB: betabloqueadores; BRA: bloqueadores dos receptores de angiotensina; DIU: diuréticos; MRPA: medida residencial da pressão arterial; PAS: pressão arterial sistólica. As diferenças são significativas quando não há sobreposição de intervalos de confiança de 95%.*

**Figura 3:**
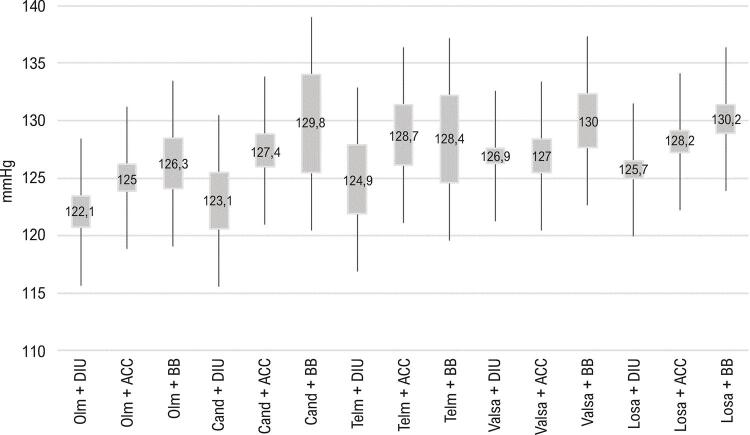
– *Comparação das médias de PAS (MRPA) obtidas com utilização dos diversos tipos de BRA em combinação dupla. ACC: antagonistas dos canais de cálcio; BB: betabloqueadores; BRA: bloqueadores dos receptores de angiotensina; Cand: candesartana; DIU: diuréticos; Losa: losartana; MRPA: medida residencial da pressão arterial; Olm: olmesartana; PAS: pressão arterial sistólica; Telm: telmisartana; Valsa: valsartana. As diferenças são significativas quando não há sobreposição de intervalos de confiança de 95%.*

A média da PAS pela MRPA com utilização de BRA + ACC e BRA + DIU foi menor do que aquela com BRA em monoterapia. Quando verificado o tipo de BRA utilizado em monoterapia, os valores de PA são progressivamente maiores com olmesartana, candesartana, telmisartana, valsartana e losartana ( Figura 2 ). Em relação às combinações, em termos gerais, os valores para a média da PAS pela MRPA são progressivamente maiores com DIU, ACC e BB, e as combinações com losartana tendem a apresentar valores maiores do que as combinações com BRAs de meia-vida mais longa ( Figura 3 ).

**Figura 4:**
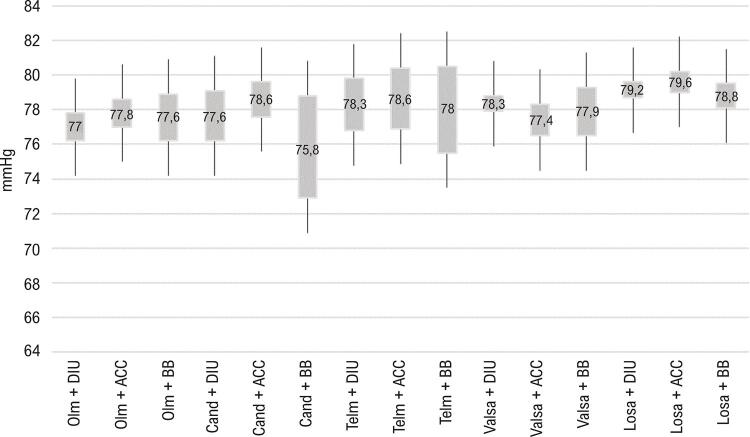
– *Comparação das médias de PAD (MRPA) obtidas com utilização de BRA (classes e tipos) em combinação dupla. ACC: antagonistas dos canais de cálcio; BB: betabloqueadores; BRA: bloqueadores dos receptores de angiotensina; Cand: candesartana; DIU: diuréticos; Losa: losartana; MRPA: medida residencial da pressão arterial; Olm: olmesartana; PAD: pressão arterial diastólica; Telm: telmisartana; Valsa: valsartana. As diferenças são significativas quando não há sobreposição de intervalos de confiança de 95%.*

**Figura 5:**
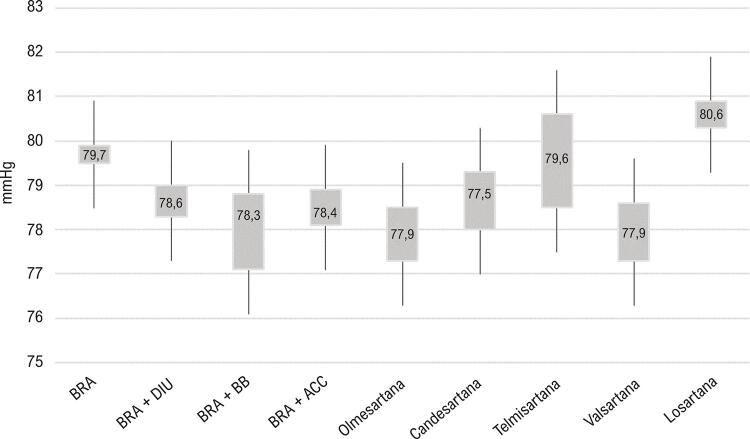
– *Comparação das médias de PAD (MRPA) obtidas com utilização de BRA (classes e tipos) em monoterapia ou em combinação dupla. ACC: antagonistas dos canais de cálcio; BB: betabloqueadores; BRA: bloqueadores dos receptores de angiotensina; DIU: diuréticos; MRPA: medida residencial da pressão arterial; PAD: pressão arterial diastólica. As diferenças são significativas quando não há sobreposição de intervalos de confiança de 95%.*

As médias de PAD foram maiores com a utilização de BRA como monoterapia quando comparadas a qualquer combinação dupla. Quanto ao tipo de BRA, em monoterapia os maiores valores médios de PAD pela MRPA foram com losartana ( Figura 4 ). Quando considerados os diversos tipos de BRA com as possíveis combinações, não houve diferença entre os valores de PAD obtidos ( Figura 5 ).

A PP foi maior na utilização de BRA + BB quando comparada às outras combinações e à BRA em monoterapia. A losartana em monoterapia ou em combinação dupla apresentou maior média de PP que a candesartana e a telmisartana.

A variabilidade da PA foi maior com uso de BRA + ACC comparado com as combinações com DIU e BB e monoterapia. A variabilidade foi menor com a telmisartana, em monoterapia ou combinação, quando comparado a valsartana. A losartana com ACC apresentou menor média de variabilidade quando comparada a outras combinações. A candesartana com BB apresentou maior variabilidade que a candesartana com ACC. Não houve diferença da variabilidade entre as diversas combinações com valsartana, olmesartana e telmisartana.

## Discussão

O presente estudo apresenta uma evolução da análise publicada em 2020 que demonstrou, em hipertensos tratados com monoterapia ou combinação dupla, médias de PAS e PAD significativamente mais baixas pela MRPA em comparação à medida casual e encontrou, nos BRAs, a opção mais utilizada no tratamento.^7^ Faz sentido, portanto, avaliar aspectos relacionados ao comportamento da PA, tanto no consultório quanto no domicílio do paciente, com os diversos fármacos que compõem a classe dos BRAs.

A amostra estudada foi constituída por uma população de idade média próxima aos 60 anos e com IMC aumentado; além disso, há predomínio do sexo feminino. Com relação à distribuição da amostra pelas regiões geográficas, há predomínio das regiões Nordeste e Sudeste. É importante considerar que os fatores idade e excesso de peso aqui encontrados podem causar maior dificuldade na obtenção das metas preconizadas para o tratamento da HA.^1 , 11 - 13^

Cabe ressaltar que ocorreu no último ano, fruto de evidências publicadas com base de dados nacional sobre a MRPA, ajuste no valor de referência para a normalidade de 135x85 mmHg para 130x80 mmHg.^1 , 14 - 16^ Essa mudança justifica a diferença encontrada nas taxas de controle pela medida casual e MRPA encontradas nesta análise em relação ao artigo publicado previamente.^7^

Em relação à estratégia de tratamento adotada nesta amostra, os BRAs foram usados em monoterapia em 48,5% dos casos; combinados com DIUs em 23,4%; com ACCs em 16,8%; e com BBs em 11,2%. Interessante notar que, a despeito de recomendações unânimes das diretrizes de hipertensão para a combinação dos fármacos na maioria dos cenários da doença hipertensiva, a monoterapia ainda permanece muito frequente.^1 - 3^ As combinações duplas com DIUs e ACCs foram preferidas, o que está bem alinhado com as recomendações atuais.^1 , 7 , 17 - 19^

Outro aspecto relevante para a escolha de fármacos no tratamento da HA é que tenham preferencialmente meia-vida longa e que permitam uma única tomada ao dia, pois essas características interferem diretamente na adesão ao tratamento e no controle adequado da PA. Para os fármacos de meia-vida curta, há que se respeitar as características farmacodinâmicas e prescrever a tomada duas ou mais vezes ao dia para que o nível plasmático e a eficácia na redução dos níveis tensionais sejam mantidos.^1 , 7 , 20 - 22^

É interessante relembrar que existem diferenças importantes, do ponto de vista farmacológico, entre esses fármacos, pois esses aspectos podem estar relacionados às diferenças que encontramos na análise de comportamento da PA, visto que os BRAs têm meias-vidas diferentes: losartana 2 h, valsartana 6 h, candesartana 9 h, olmesartana 12h e telmisartana 24 h.^23^

Quando avaliamos a taxa de controle da PA pela medida casual e pela MRPA, encontramos 56,3% e 44,5% dentro das metas, respectivamente, e, ao analisarmos o comportamento da PA com os diferentes BRAs e também com as diferentes combinações duplas, encontramos diferenças no percentual de pacientes controlados.

Para uma análise mais refinada desse comportamento, realizamos (pela MRPA) uma avaliação das médias e do intervalo de confiança de PAS e PAD, assim como da variabilidade pressórica. Em relação à estratégia de combinação dos BRAs com outras classes de anti-hipertensivos, a combinação com BB apresentou valores mais altos para a média e variabilidade da PAS em comparação à combinação com DIU ou ACC. Entre os diferentes tipos de BRA em monoterapia, a losartana apresenta os valores médios de PAS e PAD mais elevados em relação aos BRA de meia-vida mais longa.

São limitações desta análise o fato de estarmos lidando com um estudo observacional, sem o detalhamento das doses empregadas em cada fármaco, e que também não é representativo da população brasileira. Por outro lado, trata-se de uma grande base de dados, e o estudo reflete as estratégias relacionadas ao uso dos BRA em pacientes hipertensos e permite obter parâmetros importantes em relação ao comportamento da PA com os diferentes fármacos em monoterapia e em combinação.

Esses achados são compatíveis com estudos randomizados previamente publicados que avaliaram a potência anti-hipertensiva dos diferentes BRAs^24 - 28^ e, ainda mais importante, refletem a necessidade de revisarmos a estratégia dos fármacos anti-hipertensivos disponibilizados na cesta básica do SUS^6^ e prescritos para o tratamento da doença hipertensiva, pois, sabidamente, pequenas diferenças na redução da PA em hipertensos têm repercussões importantes na morbimortalidade cardiovascular.

## Conclusões

Em hipertensos tratados com BRAs, a estratégia de monoterapia ainda é frequente e, quando combinados, a opção pelos DIUs e pelos ACCs é a preferida.

Dentre os BRAs, a losartana em monoterapia e combinação dupla ainda é utilizada em mais da metade dos pacientes, apesar de ser a menos eficiente na redução e no controle da PA.

Existem claras diferenças na meia-vida entre os fármacos que compõem a classe dos BRAs, percebidas no comportamento da PA avaliado tanto pela medida casual quanto pela MRPA. Essas diferenças podem refletir na efetividade do controle pressórico.

### Co-investigadores nacionais

Adriana Siqueira Serpa de Menezes, SAVE, Recife, PE. Andréa Araújo Brandão, Universidade do Estado do Rio de Janeiro, RJ. Anibal Prata Barbosa, Prog de Hip. Arterial Secretaria de Saúde de Duque de Caxias, RJ. Antonio Almeida Braga, PROCAPE, UPE, Recife, PE. Antonio Eduardo de Melo Filho, Clínica de Saúde Dr Antonio Eduardo de Melo, Triunfo, PE. Átila de Oliveira Melo, Liga de Hipertensão Arterial UFG, Goiânia, GO. André K Vidigal de Vasconcellos, Instituto de Cardiologia do Agreste, Caruaru, PE. Audes D. M. Feitosa, Unidade de Hipertensão e Cardiologia Preventiva, PROCAPE/UPE, Recife, PE. Breno Gontijo de Camargos, AngioCor, Taguatinga, DF. Bruno Alencar Fonseca, Clínica Blues, Belo Horizonte, MG. Bruno Daniel Ferrari, Fundação Educacional do Município de Assis, FEMA, SP. Bruno José Peixoto Coutinho, Cardiologia Hospital Oswaldo Cruz, Universidade de Pernambuco, PE. Carlo Bonasso, Clínica Médica Carlo Bonasso SS Ltda, São Paulo, SP. Carlos José Mota de Lima, Centro Cardiológico São Camilo, CE. Carlos Filinto de Almeida, Instituto do Coração de Mato Grosso do Sul, Campo Grande, MS. Claudinelli Alvarenga Aguilar, Clínica do Esporte, Goiânia, GO. César Ricardo Soares Medeiros, Clínica de Cardiologia Dr César Medeiros, Ribeirão Preto, SP. Cristiano Pederneiras Jaeger, Instituto de Medicina Vascular - Coracentro, Porto Alegre/RS. Daniel Lages Dias, Novacordis, Paulínia, SP. Diogo da Silva Amorim, Liga de Hipertensão Arterial UFG, Goiânia, GO. Ednaldo M. Fontes Segundo, Cardiologista pela SBC, Instituto Paulo Gomes (IPG) em Estância, SE. Eduardo C. D. Barbosa, Dept Hipertensão e Cardiometabolismo Hospital São Francisco, Santa Casa Porto Alegre, RS. Eduardo Érico Zen, Hospital Cardiológico Costantini, Curitiba, PR. Elder Gil A. Cruz, Clínica do Coração Dr. Elder Gil, Salgueiro, PE. Esther G. Diôgo de Lima de B. Carvalho, Clínica São Lucas, Guarabira, PB. Fábio Argenta, Mediodonto, Cuiabá, MT. Fabiano de Souza Ramos, MEDCOR Cardiologia, Nova Iguaçu- RJ. Flávia Karina Silva e Oliveira, Centro de Cardiologia, São José dos Campos, SP. Flávio H. A. P. Véras, Clínica do Coração, Mossoró, RN. Francisco Deoclecio Pinheiro, Clínica de Especialidades Médicas de Itapipoca, Itapipoca, CE. Frank Land L. de Carvalho, Cardiovasf, Petrolina, PE. Germano Granja, Clínica do Coração, Ouricuri, PE. Giovanni Saraiva, Imedi e Icordis, Recife, PE. Gleidson Junio Oliveira de Souza, Liga de Hipertensão Arterial UFG, Goiânia, GO. Gustavo Barros - MCOR / Recife-PE. Gustavo Guimarães Moreira de Castro, ITACORDIS e Universidade Iguaçu – UNIG, Itaboraí e Nova Iguaçu, RJ. Jadil Francisco Fusturath Júnior, Cardio Service, Porto Velho, RO. José Wladimir Tambelli Pires, Clínica de Cardiologia, Itapetininga/SP. João Evaristo de Oliveira Dantas, Cardiomed/Multimed, São Luís, MA. João Félix de Morais Filho, Clínica Angiocárdio, Natal / RN. João Francisco Martins Pacheco, Endocardio, Belém, PA. Jonathan Scapin Zagatti, Cardio Ritmo Diagnósticos, Jales, SP. José Joaquim Raposo, Serviço de Cardiologia da Santa Casa de Limeira, SP. José Roberto Moya, Biocardios, Cuiabá, MT. Josafá de Oliveira Costa, Clínica Vitta, Igarassu, PE. Josiedson Pontes de Farias, Cardio Diagnósticos, Caruaru, PE. Juan Carlos Yugar Toledo. Endocor, Rio Preto, SP. Lilian Mesquita, Ergo Med Setor De Cardiologia/Geriatria, RJ. Lola Helbingen Santos, Cardiodiagnósticos, Goiania, GO. Luam Vieira de Almeida Diógenes, Procardiaco, Teresina, PI. Luiz Kencis Júnior, Lapacor, São Paulo, SP. Marcelo Júlio de Oliveira, Clínica Cardiograficos, Ribeirão Preto, SP. Marco Antônio de M Alves, Escada Clinical Center, Escada, PE. Marco A. M. Gomes, Centro de Pesquisas Clínicas do Cesmac/Hospital do Coração de Alagoas. Marcos Alberto Pires Meira Júnior, Clincar João Pessoa PB. Maria Christina Cavalcanti Ballut, MEDCENTRO, Manaus, AM. Marcus Vinícius de Oliveira, Cardiodiagnósticos, Goiânia-GO. Maria Beatriz M. B. L. Rodrigues, Cardiovida, Porto Velho, RO. Mayara Cedrim Santos, Instituto UNICAP de Pesquisa Clínica, Recife, PE. Naiara Pedrassi Engracia Garcia Caluz, Centro de Medicina Avançada Dr Luiz Kencis, São Paulo, SP. Nelson Dinamarco, Ambulatório de Hipertensão Arterial, Colegiado de Medicina, Universidade Estadual Santa Cruz – UESC. Nildo Magalhães, SOBAM Jundiaí, SP. Paulo Roberto Pereira de Sant’Ana, MEDCOR Cardiologia, Nova Iguaçu- RJ. Rafael Nogueira de Macedo, Centro Cardiológico São Camilo, CE. Paulo Sérgio Lopes Soares, Universidade De Vassouras - Hospital Universitário de Vassouras, RJ. Ricardo Mesquita de Freitas, Cardiocenter, Barreiras, BA. Roberto de A. Dultra, Clinicor, Itabuna, Bahia, BA. Roberto Dischinger Miranda, Serviço de Cardiologia, Disciplina de Geriatria e Gerontologia, Escola Paulista de Medicina, Universidade Federal de São Paulo. Rodrigo Cunha de Sousa, Centro Integrado de Medicina Invasiva – CIMI, Uberaba, MG. Rogério Krakauer, Santa Casa de SP. Rogerio Ruiz, HD HomeDoctor, SP. Ruy Morando, Cincor - Centro Integrado do Coração, Americana, SP. Sérgio Augusto Vieira Simõe, Consultório Médico Integrado, Tássia Tâmara Silva Feitosa, Ok Doutor, Recife, PE. Tobias Barreto, SE. Sheyla Cristina Tonheiro Ferro da Silva, CLINSAUDE e CEMISE, Aracaju, SE. Vanderlei Magalhães da Silveira, Cardiologista, Faculdade de Medicina, Universidade de Passo Fundo. Vanildo Guimarães, Diagnóstico Cardíaco, Recife, PE. Vilma Helena Burlamaqui, Consultório de Cardiologia, Niterói, RJ. Vitor Bruno Teixeira de Holanda, Climile, Ananindeua, PA. Walmir de Vasconcelos Ratier Thomaz, Rio de Janeiro, RJ. Weimar Sebba Barroso, Liga de Hipertensão Arterial UFG, Goiânia, GO. Wenderson Tavares dos Santos, Hospital Biocor, Belo Horizonte, MG
