# Supplementary material for: Bloqueadores do Receptor de Angiotensina Avaliados por Medida de Consultório e Residencial da Pressão Arterial. Estudo TeleMRPA
Source: Arq Bras Cardiol. 2022 Apr 26;118(6):1069–82. [Article in Portuguese] doi: 10.36660/abc.20210504 (PMC9345159; doi:10.36660/abc.20210504)
Supplement: Translation

# Translation: Angiotensin Receptor Blockers Evaluated by Office and Home Blood Pressure Measurements. TeleHBPM Study

**Authors:** Weimar Kunz Sebba Barroso, Andréa Araujo Brandão, Priscila Valverde de Oliveira Vitorino, Audes Diógenes de Magalhães Feitosa, Eduardo Costa Duarte Barbosa, Roberto Dischinger Miranda, Josep Redon, Miguel Camafort-Babkowski, Antonio Coca, Marco Antônio Mota Gomes

**Affiliations:** 1 Brazil Universidade Federal de Goiás - Liga de Hipertensão Arterial, Goiânia, GO – Brazil; 2 Brazil Universidade do Estado do Rio de Janeiro – Cardiologia, Rio de Janeiro, RJ – Brazil; 3 Brazil Pontifícia Universidade Católica de Goiás - Escola de Ciências Sociais e da Saúde,Goiânia, GO – Brazil; 4 Brazil Universidade de Pernambuco, Recife, PE – Brazil; 5 Brazil Universidade Católica de Pernambuco, Recife, PE – Brazil; 6 Brazil Complexo Hospitalar Santa Casa de Misericórdia de Porto Alegre – Cardiologia, Porto Alegre, RS – Brazil; 7 Brazil Universidade Federal de São Paulo - Escola Paulista de Medicina, São Paulo, SP – Brazil; 8 Spain University of Valencia – Hypertension, Valencia, Comunitat Valenciana – Spain; 9 Spain University of Barcelona - Hospital Clínic. Hypertension Unit, Barcelona, Catalunya – Spain; 10 Spain Hypertension and Vascular Risk Unit. Hospital Clinic. University of Barcelona,Barcelona – Spain; 11 Brazil Centro Universitário CESMAC - Hospital do Coração, Maceió, AL – Brazil

**Keywords:** Hypertension, Angiotensin II Type 1 Receptor Blockers, Losartana, Antihypertensive Agents/therapeutic use, Age, Sex, Body Weights and Measures

## Abstract

**Background:**

Adequate treatment of arterial hypertension and achieving arterial hypertension goals in are important in reducing cardiovascular outcomes.

**Objectives:**

To describe angiotensin receptor blockers in monotherapy or double combination therapy and the rate of arterial hypertension control.

**Methods:**

This cross-sectional study evaluated patients who were using angiotensin receptor blockers between 2017 and 2020. Those using three or more antihypertensive drugs were excluded. The analyzed variables included sex, age, body mass index, valid home blood pressure monitoring (HBPM) measurements, casual and HBPM systolic and diastolic blood pressure measurements, blood pressure variability, and antihypertensive and angiotensin receptor blocker class. Paired t, chi-square, and Fisher’s exact tests were used, as well as overlapping 95% confidence intervals and a significance level of 5% (p < 0.05).

**Results:**

Of 17,013 patients, 12,813 met the inclusion criteria, 62.1% of whom were female. The mean number of valid measurements was 23.3 (SD, 2.0). The mean HBPM and casual measurements for systolic blood pressure were 126.8 (SD, 15.8) mmHg and 133.5 (SD, 20.1) mmHg (p <0.001), respectively, while those for diastolic blood pressure were 79.1 (SD, 9.7 mmHg) and 83.6 (SD, 11.9) mmHg (p <0.001), respectively. Losartan was the most common angiotensin receptor blocker and resulted in the highest blood pressure values. Combinations of angiotensin receptor blockers with diuretics or calcium channel antagonists resulted in lower blood pressure values.

**Conclusions:**

More than half of the patients used losartan, although it was the least efficient drug for reducing and controlling blood pressure.

## Introduction

Adequate treatment and control is one of the great challenges in arterial hypertension, which is the leading cause of death worldwide. Aligning treatment strategies with the most current scientific is one way to optimize these results.^1 - 3^ Drugs that effectively reduce blood pressure (BP) also protect against the main outcomes of hypertensive disease, and the best results can be expected of drugs with a long half-life (thus, a single daily dose) that do not negatively interfere in metabolic parameters. It is also known that small BP reductions, even in the early stages of arterial hypertension, can lead to reductions in the main cardiovascular outcomes.^1 , 4 , 5^

On the other hand, despite such evidence, the Brazilian Unified Health System provides medications with a short half-life that are used in monotherapy and require several doses a day. Such characteristics can negatively impact adherence and hinder adequate BP control. It should be emphasized that the Brazilian Unified Health System reflects the drug strategy used for 75% of the hypertensive patients in our country.^1 , 6^

A 2021 study evaluated a database of 22,446 individuals who underwent home and office BP measurement, 11,337 of whom were being treated for hypertension by cardiologists with antihypertensive drugs. In 74.6% of the cases, renin-angiotensin-aldosterone system blockade was used, including angiotensin receptor blockers (ARBs) in 58.7%, either in monotherapy or combination therapy.^7^

The objectives of the present study were: (i) to verify the distribution of ARB prescription in monotherapy and combined therapy according to sex, geographic region, and diabetes status; (ii) to compare BP control according to casual and home BP monitoring measurement (HBPM) for all ARB treatment strategies; (iii) to compare BP control in casual and HBPM measurements; and (iv) to compare mean systolic blood pressure (SBP), diastolic blood pressure (DBP), pulse pressure (PP), and BP variability obtained through ARBs in monotherapy or double combination therapy, considering the class as a whole and individual types.

## Methods

This study was approved by the Human Research Ethics Committee of the Hospital das Clínicas of the Federal University of Goiás (opinion 99691018.7.0000.5078) and evaluated patients who were examined on the TeleHBPM platform (www.telemrpa.com) between May 2017 and October 2020.

The platform was developed as a remote reporting tool for telemonitoring, including features that allow the database to be analyzed and filtered according to research questions. The mathematical algorithm allows analysis while protecting the personal data of patients and health facilities, whether interpreting exams or developing research projects. Since it is not software, but a platform accessible on any device via an Internet connection, BP measurements can be uploaded quickly and remotely.^8^

The database search was limited to patients who used ARBs. Patients aged at least aged 18 years on monotherapy or double combination therapy were included. Patients on a combination of three or more antihypertensives, antihypertensives in combination with angiotensin-converting enzyme inhibitors, or antihypertensives in double combination therapy with infrequently used antihypertensives (eg, spironolactone, direct vasodilators, alpha2 agonists) were excluded ( Figure 1 ). We also excluded irbesartan from the results due to its rarity in the overall sample.

**Figure 1:** – *Sample selection flowchart. ARB: angiotensin receptor blockers; ACEI: angiotensin-converting enzyme inhibitors.*

The following data were collected from the TeleHBPM platform: sex, age (in years), body mass index, number of valid HBPM measurements, casual and HBPM SBP and DBP measurements, blood pressure variability based on HBPM measurements obtained through the standard deviation of the 24 household measurements taken during the protocol, drug class used, and type of ARB. The regional distribution of the sample was also evaluated, as was the prevalence of individuals who used medications to treat diabetes mellitus (oral antidiabetics and/or insulin).

The Quetelet formula was used to calculate body mass index based on weight and height data.^9^ HBPM was performed with the provided device; patients were instructed about proper handling and BP measurement on the day the device was delivered.^1^ On that day, two measurements were taken in a clinical/office environment and, over the next 4 days, the patient (and/or caregiver/companion) performed the measurements at home according to protocol. The mean of the two measurements taken on the first day was considered the casual measurement, and the mean of the 24 measurements taken from the second to the fifth day was considered the HBPM measurement.^8 , 10^ Only validated automatic devices (Omron, Geratherm, and Microlife) were used.

The data were exported from the TeleHBPM platform to Microsoft Excel. All drug classes described on the platform were reviewed and coded by two work teams. The databases were then cross-referenced to identify discrepant data, which, when present, were reviewed by the entire team. Individuals whose SBP/DBP values were <140/90 mmHg in casual measurement and <130/80 mmHg in HBPM, respectively, were considered to have controlled BP.^1^

### Statistical analysis

Statistical analysis was performed in Stata 14.0. Quantitative variables were expressed as mean and standard deviation, and qualitative variables were expressed as absolute and relative frequencies. The Kolmogorov-Smirnov test was used to verify the normality of the data.

The mean SBP, DBP and PP values obtained in casual and HBPM measurements were compared using a paired Student’s *t* -test. The chi-square test or Fisher’s exact test was used to compare BP control rates according to the casual and HBPM measurements, as well as to compare the rates of BP control for each drug strategy.

Overlapping 95% confidence intervals were used to compare the differences in mean SBP, DBP, PP and BP variability obtained with ARB monotherapy or double combination therapy, considering the class as a whole and individual types. P-values <0.05 were considered significant.

## Results

A total of 12,813 patients were evaluated, the majority of whom were female. The Northeast was the most prominently represented region, with approximately half of the patients. The prevalence of diabetes was 6.2% ( Table 1 ).

**Table 1:** – Description of hypertensive patients using ARBs, n = 12,813

Variable	Total n (%)	ARB n (%)	ARB + DIU n (%)	ARB + BB n (%)	ARB + CCA n (%)
	**12,813 (100)**	**6225 (48.6)**	**3006 (23.5)**	**1433 (11.2)**	**2.149 (16,8)**
**Sex**					
Female	7953 (62.1)	3749 (60.2)	2006 (66.7)	980 (68.4)	1218 (56.7)
Male	4860 (37.9)	2476 (39.8)	1000 (33.2)	453 (31.6)	931 (43.3)
**Region**					
Unidentified	37 (0.3)	12 (0.2)	16 (0.5)	5 (0.3)	4 (0.1)
Northeast	6347 (49.6)	3187 (51.2)	1355 (45.1)	698 (48.7)	1107 (51.5)
North	802 (6.3)	326 (5.2)	194 (6.5)	52 (3.6)	230 (10.7)
Midwest	1003 (7.8)	478 (7.7)	232 (7.7)	162 (11.3)	131 (6.1)
Southeast	4028 (31.4)	1961 (31.5)	1026 (34.1)	444 (31.0)	597 (27.8)
South	596 (4.7)	261(4.2)	183 (6.1)	72 (5.0)	80 (37)
**Diabetes**					
No	12,015 (93.8)	5877 (94.4)	2811 (93.5)	1294 (90.3)	2033 (94.6)
Yes	798 (6.2)	348 (5.6)	195 (6.5)	139 (9.7)	116 (5.4)

*CCA: calcium channel antagonists; BB: beta-blockers; ARB: angiotensin receptor blockers; DUI: diuretics.*

Double combination therapy was slightly more prevalent than monotherapy (51.2% vs. 48.5%). The following types of ARBs were used: losartan (57.2%), olmesartan (18.8%), valsartan (15.0%), telmisartan (4.8%), candesartan (3.8%), and irbesartan (0.4%).

The mean number of valid HBPM measurements was 23.3(SD, 2.0). The differences in mean casual and HBPM values for SBP and DBP were 6.7 mmHg (p < 0.001) and 4.5 mmHg (p < 0.001), respectively. These differences characterize the white-coat effect and were maintained across all treatment strategies. This behavior was repeated in all ARBs, whether in monotherapy or combination therapy. We also compared the rate of BP control by casual and HBPM measurements in monotherapy and combination therapy ( Table 2 ).

**Table 2:** – Sample description and comparison of blood pressure control by casual measurement and by HBPM according to the use of ARB in monotherapy and combinations, n = 12,813

Variable	HBPM	Casual	p*
**Total (n = 12,813)**			
SBP	126.8±15.8	133.5±20.1	< 0.001
DBP	79.1±9.7	83.6±11.9	< 0.001
PP	52.2±14.4	49.9±16.1	< 0.001
**ARB monotherapy (n = 6225)**			
SBP	126.9±15.6	133.5±19.8	< 0.001
DBP	79.7±9.6	84.3±11.7	< 0.001
PP	51.7±14.0	49.215.7	< 0.001
**ARB + DIU (n = 3006)**			
SBP	125.0±15.8	132.3±20.3	< 0.001
DBP	78.6±9.5	83.3±11.9	< 0.001
PP	50.7±14.3	49.1±16.1	< 0.001
**ARB + CCA (n = 2149)**			
SBP	127.0±14.9	133.8±19.2	< 0.001
DBP	78.4±9.9	82.8±11.9	< 0.001
PP	53.2±14.0	51.0±15.8	< 0.001
**ARB + BB (n = 1433)**			
SBP	129.4±17.9	136.0±22.2	< 0.001
DBP	78.3±10.4	82.6±12.4	< 0.001
PP	56.016.2	53.417.7	< 0.001
**Variable**	**Controlled**	**Not controlled**	p**
**Total**			
HBPM	5695 (44.5)	7118 (55.5)	< 0.001
Casual measurement	7211 (56.3)	5602 (43.7)	
**ARB monotherapy**			
HBPM	2691 (43.2)	3534 (56.8)	0.007
Casual measurement	3485 (56.0)	2740 (44.0)	0.513
**ARB + DIU**			
HBPM	1441 (48.0)	1565 (52.1)	< 0.001
Casual measurement	1751 (58.3)	1255 (41.7)	0.013
**ARB + CCA**			
HBPM	960 (44.7)	1189 (55.3)	0.818
Casual measurement	1204 (56.0)	945 (44.0)	0.796
**ARB + BB**			
HBPM	603 (42.1)	830 (57.9)	0.056
Casual measurement	771 (53.8)	662 (46.2)	0.045

*Paired t-test; **Chi-square or Fisher’s exact test. CCA: calcium channel antagonists; BB: beta-blockers; ARB: angiotensin receptor blockers; DUI: diuretics; HBPM: home blood pressure monitoring; BPD: diastolic blood pressure; SBP: systolic blood pressure; PP: pulse pressure.

Table 3 describes the mean casual and HBPM BP values and the BP control rate with different ARBs in monotherapy, while Tables 4 , 5 and 6 compare these values for ARBs combined with diuretics, calcium channel antagonists (CCA), and beta-blockers, respectively.

**Table 3:** – Sample description and comparison of blood pressure control in casual and HBPM measurements according to ARB type in monotherapy, n = 6225

Variable	HBPM	Casual measurement	p*
**Losartan (n = 3.861)**			
SBP	128.3 ±15.8	135.4± 20.3	< 0.001
DBP	80.6±9.7	85.5±11.8	< 0.001
PP	52.1±14.1	50.0±16.0	< 0.001
**Valsartan (n = 818)**			
SBP	126.8±15.3	132.4±19.5	< 0.001
DBP	78.6±9.5	82.4±10.8	< 0.001
PP	52.7±14.3	50.0±16.0	< 0.001
**Candesartan (n = 221)**			
SBP	124.0±12.9	129.0±17.0	< 0.001
DBP	77.5±7.8	81.4±9.5	< 0.001
PP	50.9±13.4	47.6±14.8	< 0.001
**Olmesartan (n = 1.032)**			
SBP	123.0±14.9	128.418.1	< 0.001
DBP	77.9±9.4	82.0±11.9	< 0.001
PP	49.8±13.0	46.414.1	< 0.001
**Telmisartan (n = 287)**			
SBP	126.2±14.8	132.6±18.0	< 0.001
DBP	79.6±9.1	84.0±11.3	< 0.001
PP	51.1±13.9	48.3±15.1	< 0.001
Variable	Controlled	Not controlled	p**
**Losartan**			
HBPM	1517 (39.3)	2344 (60.7)	< 0.001
Casual	1984 (51.4)	1877 (48.6)	< 0.001
**Valsartan**			
HBPM	369 (45.1)	449 (54.9)	0.693
Casual	489 (59.8)	329 (40.2)	0.037
**Candesartan**			
HBPM	111 (50.2)	110 (49.8)	0.081
Casual	150 (67.9)	71 (32.1)	< 0.001
**Olmesartan**			
HBPM	559 (54.2)	473 (45.8)	< 0.001
Casual	682 (66.1)	350 (33.9)	< 0.001
**Telmisartan**			
HBPM	130 (45.3)	157 (54.7)	0.770
Casual	172 (59.9)	115 (40.1)	0.207

*Paired t-test; **Chi-square or Fisher’s exact test. ARB: angiotensin receptor blockers; HBPM: home blood pressure monitoring; BPD: diastolic blood pressure; SBP: systolic blood pressure; PP: pulse pressure.

**Table 4:** – Comparison of blood pressure control in casual and HBPM measurement according to ARB type in double combination therapy with DUIs, n = 3006

Variable	HBPM	Casual measurement	p
**Olmesartan + DIU (n = 530)**			
SBP	122.1±15.8	128.4±20.2	< 0.001
DBP	77.0±9.6	81.112.0	< 0.001
PP	49.5±15.1	47.3±16.3	< 0.001
**Candesartan + DIU (n = 151)**			
SBP	123.1±5.0	130.920.8	< 0.001
DBP	77.6±9.1	82.4±12.1	< 0.001
PP	49.6±14.1	48.5±15.1	0.199
**Telmisartan + DIU (n = 123)**			
SBP	124.9±16.7	132.5±20.1	< 0.001
DBP	78.3±8.5	83.6±11.1	< 0.001
PP	51.1±15.9	48.9±16.8	< 0.001
**Valsartan + DIU (n = 1.920)**			
SBP	126.9±15.5	132.7±20.1	< 0.001
DBP	78.3±9.7	82.1±11.7	< 0.001
PP	53.2±14.3	50.6±16.1	< 0.001
**Losartan + DIU (n = 1.715)**			
SBP	125.7±15.7	133.8±20.1	< 0.001
DBP	79.2±9.4	84.2±11.7	< 0.001
PP	50.9±14.1	49.6±16.1	< 0.001
Variable	Controlled	Not controlled	p**
**Olmesartan + DIU**			
HBPM	288 (54.3)	242 (45,7)	< 0,001
Casual	335 (63.2)	195 (36,8)	0,001
**Candesartan + DIU**			
HBPM	80 (53.0)	71 (47,0)	0,034
Casual	99 (65.6)	52 (34,4)	0,021
**Telmisartan + DIU**			
HBPM	59 (48.0)	64 (52,0)	0,430
Casual	73 (59.4)	50 (40,6)	0,490
**Valsartan + DIU**			
HBPM	887 (46.2)	1.033 (53,8)	0,094
Casual	1.136 (59.2)	784 (40,8)	0,006
**Losartan + DIU**			
HBPM	779 (45.4)	936 (54,6)	0,382
Casual	965 (56.3)	750 (43,7)	0,992

*Paired t-test; **Chi-square or Fisher’s exact test. ARB: angiotensin receptor blocker; DUI: diuretic; HBPM: home blood pressure monitoring; BPD: diastolic blood pressure; SBP: systolic blood pressure; PP: pulse pressure.

**Table 5:** – Sample description and comparison of blood pressure control in casual and HBPM measurement according to ARB type in double combination therapy with CCAs, n = 2,149

Variable	HBPM	Casual measurement	p*
**Olmesartan + CCA (n = 626)**			
SBP	125.0±14.9	131.7±19.4	< 0.001
DBP	77.8±10.2	81.8±12.5	< 0.001
PP	51.9±14.5	49.9±15.9	< 0.001
**Candesartan + CCA (n = 419)**			
SBP	127.4±14.6	135.1±18.4	< 0.001
DBP	78.6±10.2	83.6±11.6	< 0.001
PP	53.4±13.9	51.5±15.4	< 0.001
**Telmisartan + CCA (n = 136)**			
SBP	128.7±15.8	132.4±18.8	0.003
DBP	78.6±10.3	81.8±11.7	< 0.001
PP	55.1±13.6	50.7±14.1	< 0.001
**Valsartan + CCA (n = 433)**			
SBP	127.0±15.2	132.6±19.5	< 0.001
DBP	77.4±9.6	80.7±11.6	< 0.001
PP	54.2±13.6	51.8±15.4	< 0.001
**Losartan + CCA (n = 903)**			
SBP	128.2±14.5	135.9±18.7	< 0.001
DBP	79.6±9.6	84.7±11.3	< 0.001
PP	53.1±3.7	51.1±15.9	< 0.001
Variable	Controlled	Not controlled	p**
**Olmesartan + CCA**			
HBPM	302 (48.2)	324 (51.8)	0.050
Casual	378 (60.4)	248 (39.6)	0.034
**Candesartan + CCA**			
HBPM	173 (41.3)	246 (58.7)	0.186
Casual	218 (52.0)	201 (48.0)	0.075
**Telmisartan + CCA**			
HBPM	69 (50.7)	67 (49.3)	0.138
Casual	84 (61.8)	52 (38.2)	0.195
**Valsartan + CCA**			
Casual	270 (62.4)	163 (37.6)	0.010
HBPM	206 (47.6)	227 (52.4)	0.183
**Losartan + CCA**			
HBPM	361 (40.0)	542 (60.0)	0.005
Casual	451 (49.9)	452 (50.1)	< 0.001

*Paired t-test; **Chi-square or Fisher’s exact test. CCA: calcium channel antagonists; ARB: angiotensin receptor blockers; HBPM: home blood pressure monitoring; BPD: diastolic blood pressure; SBP: systolic blood pressure; PP: pulse pressure.

**Table 6:** – Sample description and comparison of blood pressure control in casual and HBPM measurement according to ARB type in double combination therapy with BBs, n = 1,433

Variable	HBPM	Casual	p*
**Olmesartan + BB (n = 230)**			
SBP	126.3±17.0	132.0±20.6	< 0.001
DBP	77.6±10.4	80.911.5	< 0.001
PP	53.6±14.8	51.1±17.0	< 0.001
**Candesartan + BB (n = 65)**			
SBP	129.8±17.3	133.8±21.0	< 0.001
DBP	75,8±11.8	79.114.2	0.012
PP	59.0±17.1	54.716.6	0.002
**Telmisartan + BB (n = 75)**			
SBP	128.4±16.5	132.6±21.9	0.01
DBP	78.0±10.7	82.0±13.9	< 0.001
PP	55.2±15.3	50.6±16.3	< 0.001
**Valsartan + BB (n = 213)**			
SBP	130.0±16.8	137.021.9	< 0.001
DBP	77.9±10.3	82.5±12.5	< 0.001
PP	57.0±15.7	54.5±18.0	< 0.001
**Losartan + BB (n = 851)**			
SBP	130.2±18.5	137.3±22.7	< 0.001
DBP	78.8±10.3	83.4±12.3	< 0.001
PP	56.2±16.7	53.8±17.9	< 0.001
Variable	Controlled	Not controlled	p**
**Olmesartan + BB**			
HBPM	114 (49.6)	116 (50.4)	0.115
Casual	138 (60.0)	92 (40.0)	0.251
**Candesartan + BB**			
HBPM	31 (47.7)	34 (52.3)	0.598
Casual	40 (61.5)	25 (38.5)	0.391
**Telmisartan + BB**			
HBPM	36 (48.0)	39 (52.0)	0.535
Casual	46 (61.3)	29 (38.7)	0.376
**Valsartan + BB**			
HBPM	91 (42.7)	122 (57.3)	0.610
Casual	113 (53.1)	100 (46.9)	0.338
**Losartan + BB**			
HBPM	331 (38.9)	520 (61.1)	0.001
Casual	433 (50.9)	418 (49.1)	0.001

*Paired t-test; **Chi-square or Fisher’s exact test. BB: beta-blockers; ARB: angiotensin receptor blockers; HBPM: home blood pressure monitoring; DBP: diastolic blood pressure; SBP: systolic blood pressure; PP: pulse pressure.

According to the goals of <140/90 mmHg (casual) and <130/80 mmHg (HBPM) recommended by current guidelines,^1^ overall BP control was better in casual measurement. In HBPM, BP control was lower in ARB monotherapy and in ARBs combined with beta-blockers. Among the ARB types used in monotherapy or combination therapy, BP control was lower with losartan and higher with long half-life ARBs. This trend was repeated in the casual measurements.

The control rates of different ARBs in combination with CCA, BB, or diuretics were lower in combinations with losartan and higher in ARBs with a long half-life in both HBPM and casual measurements. In HBPM, the mean SBP for ARB + CCA and ARB + diuretics was lower than that of ARB monotherapy. In monotherapy, the BP values were progressively higher for olmesartan, candesartan, telmisartan, valsartan and losartan ( Figure 2 ). In combined therapy, the mean SBP values for HBPM were progressively higher with diuretics, CCA and BB, and combinations with losartan tended to have higher values than those with longer half-life ARBs ( Figure 3 ). The mean DBP measurements were higher in ARB monotherapy than any double combination therapy. In HBPM, the ARB type with the highest mean DBP values in monotherapy was losartan ( Figure 4 ). No difference was found in DBP values between the different possible combinations of ARB types ( Figure 5 ).

**Figure 2:** – *Comparison of mean SBP (HBPM) obtained using ARB (classes and types) in monotherapy or in double combination therapy. CCA: calcium channel antagonists; BB: beta-blockers; ARB: angiotensin receptor blockers; DUI: diuretics; HBPM: home blood pressure monitoring; SBP: systolic blood pressure. Differences are significant when 95% confidence intervals do not overlap.*

**Figure 3:** – *Comparison of mean SBP (HBPM) obtained using different types of ARB in double combination therapy. CCA: calcium channel antagonists; BB: beta-blockers; ARB: angiotensin receptor blockers; Cand: candesartan; DUI: diuretics; Losa: losartan; HBPM: home blood pressure monitoring; Olm: olmesartan; SBP: systolic blood pressure; Telm: telmisartan; Valsa: valsartan. Differences are significant when 95% confidence intervals do not overlap.*

**Figure 4:** – *Comparison of mean DBP (HBPM) obtained using ARB (classes and types) in double combination therapy. CCA: calcium channel antagonists; BB: beta-blockers; ARB: angiotensin receptor blockers; Cand: candesartan; DUI: diuretics; Losa: losartan; HBPM: home blood pressure monitoring; Olm: olmesartan; DBP: diastolic blood pressure; Telm: telmisartan; Valsa: valsartan.Differences are significant when 95% confidence intervals do not overlap.*

**Figure 5:** – *Comparison of mean DBP (HBPM) obtained using ARB (classes and types) in monotherapy or in double combination therapy. CCA: calcium channel antagonists; BB: beta-blockers; ARB: angiotensin receptor blockers; DUI: diuretics; HBPM: home blood pressure monitoring; DBP: diastolic blood pressure. Differences are significant when 95% confidence intervals do not overlap.*

PP was higher with ARB + BB than any other combination or ARB monotherapy. Losartan in monotherapy or in double combination therapy resulted in a higher mean PP than candesartan or telmisartan.

BP variability was greater with ARB + CCA than in combinations with diuretics or BB or in monotherapy. Whether in monotherapy or combination therapy, BP variability was lower with telmisartan than valsartan. Losartan + CCA had lower mean variability than other combinations. Candesartan + BB showed greater variability than candesartan + CCA. There was no difference in BP variability between combinations with valsartan, olmesartan and telmisartan

## Discussion

The present study, a further development of an analysis published in 2020, found that, in hypertensive patients treated with monotherapy or double combination therapy, different possible combinations of ARB types resulted in significantly lower mean SBP and DBP in HBPM than in casual measurements, as well as that ARBs were the most common treatment option.^7^ Thus, it makes sense to assess BP behavior in response to various ARB types in both clinical and home settings.

Our sample population had a mean age of approximately 60 years and a high body mass index. The patients were also predominantly women, and most resided in the Northeast and Southeast regions. It is important to consider that advanced age and excess weight can impede achieving recommended arterial hypertension treatment goals.^1 , 11 - 13^

It should also be noted that in the last year, as a result of HBPM evidence published in the national database, the reference values for normality were lowered from 135/85 mmHg to 130/80 mmHg.^1 , 14 - 16^ This change explains the difference in BP control rates found in casual and HBPM measurements in this analysis compared to our previous article.^7^

Regarding the treatment strategies used in this sample, 48.5% received ARB monotherapy, 23.4% received ARBs combined with diuretics, 16.8% received ARBs combined with CCAs, and 11.2% received ARBs combined with BBs. Interestingly, although hypertension guidelines unanimously recommend drug combinations for most cases of hypertension, monotherapy was still quite frequent.^1 - 3^ Dual combination therapy with diuretics and CCAs was preferred, which is in line with current recommendations.^1 , 7 , 17 - 19^

Another relevant aspect in selecting arterial hypertension drugs is a long half-life, which allows a single daily dose; these characteristics directly interfere with treatment adherence and adequate BP control. Drugs with a short half-life must be taken twice or more daily to maintain their plasma level and efficacy in reducing BP levels.^1 , 7 , 20 - 22^

It is interesting to note that, from a pharmacological point of view, there are important differences between these drugs, and the different half-lives of ARBs (losartan, 2 h; valsartan, 6 h; candesartan, 9 h; olmesartan, 12 h; and telmisartan, 24 h) may be related to the differences we found in BP behavior.^23^

When evaluating the BP control rate by casual and HBPM measurements, we found that 56.3% and 44.5% of the patients, respectively, were within the goals. We found different percentages of patients with controlled BP among the different ARB types and combinations.

For a more refined analysis of this behavior, we determined the mean HBPM measurements and confidence intervals of SBP, DBP, and pressure variability. Combinations with BBs resulted in higher mean SBP values and variability than combinations with diuretics or CCAs. In monotherapy, losartan had the highest mean SBP and DBP values of the longer half-life ARBs.

This observational study was limited by the fact that it did not assess the dosage of each drug, and the sample was not representative of the Brazilian population. On the other hand, it analyzed data from a large database that reflected ARB usage strategies in hypertensive patients, allowing important parameters to be determined regarding BP behavior with different drugs in monotherapy and combination therapy.

These findings are consistent with those of previously published randomized studies that evaluated the antihypertensive efficacy of different ARBs^24 - 28^ and, more importantly, they reflect the need to review the Brazilian Unified Health System’s strategy for antihypertensive drugs,^6^ since it is known that small BP reductions in hypertensive patients have important repercussions on cardiovascular morbidity and mortality.

## Conclusions

In hypertensive patients treated with ARBs, monotherapy is still frequent. In combined therapy, diuretics and CCAs are preferred. Among ARBs, losartan is still used in more than half of patients, whether in monotherapy or double combination therapy, despite being the least efficient medication for reducing and controlling BP. There are clear differences in the half-life of ARBs, which was seen in BP behavior through both casual and HBPM measurements. These differences may reflect the effectiveness of blood pressure control.

### National co-investigators

Adriana Siqueira Serpa de Menezes, SAVE, Recife, PE. Andréa Araújo Brandão, Universidade do Estado do Rio de Janeiro, RJ. Anibal Prata Barbosa, Prog de Hip. Arterial Secretaria de Saúde de Duque de Caxias, RJ. Antonio Almeida Braga, PROCAPE, UPE, Recife, PE. Antonio Eduardo de Melo Filho, Clínica de Saúde Dr Antonio Eduardo de Melo, Triunfo, PE. Átila de Oliveira Melo, Liga de Hipertensão Arterial UFG, Goiânia, GO. André K Vidigal de Vasconcellos, Instituto de Cardiologia do Agreste, Caruaru, PE. Audes D. M. Feitosa, Unidade de Hipertensão e Cardiologia Preventiva, PROCAPE/UPE, Recife, PE. Breno Gontijo de Camargos, AngioCor, Taguatinga, DF. Bruno Alencar Fonseca, Clínica Blues, Belo Horizonte, MG. Bruno Daniel Ferrari, Fundação Educacional do Município de Assis, FEMA, SP. Bruno José Peixoto Coutinho, Cardiologia Hospital Oswaldo Cruz, Universidade de Pernambuco, PE. Carlo Bonasso, Clínica Médica Carlo Bonasso SS Ltda, São Paulo, SP. Carlos José Mota de Lima, Centro Cardiológico São Camilo, CE. Carlos Filinto de Almeida, Instituto do Coração de Mato Grosso do Sul, Campo Grande, MS. Claudinelli Alvarenga Aguilar, Clínica do Esporte, Goiânia, GO. César Ricardo Soares Medeiros, Clínica de Cardiologia Dr César Medeiros, Ribeirão Preto, SP. Cristiano Pederneiras Jaeger, Instituto de Medicina Vascular - Coracentro, Porto Alegre/RS. Daniel Lages Dias, Novacordis, Paulínia, SP. Diogo da Silva Amorim, Liga de Hipertensão Arterial UFG, Goiânia, GO. Ednaldo M. Fontes Segundo, Cardiologista pela SBC, Instituto Paulo Gomes (IPG) em Estância, SE. Eduardo C. D. Barbosa, Dept Hipertensão e Cardiometabolismo Hospital São Francisco, Santa Casa Porto Alegre, RS. Eduardo Érico Zen, Hospital Cardiológico Costantini, Curitiba, PR. Elder Gil A. Cruz, Clínica do Coração Dr. Elder Gil, Salgueiro, PE. Esther G. Diôgo de Lima de B. Carvalho, Clínica São Lucas, Guarabira, PB. Fábio Argenta, Mediodonto, Cuiabá, MT. Fabiano de Souza Ramos, MEDCOR Cardiologia, Nova Iguaçu- RJ. Flávia Karina Silva e Oliveira, Centro de Cardiologia, São José dos Campos, SP. Flávio H. A. P. Véras, Clínica do Coração, Mossoró, RN. Francisco Deoclecio Pinheiro, Clínica de Especialidades Médicas de Itapipoca, Itapipoca, CE. Frank Land L. de Carvalho, Cardiovasf, Petrolina, PE. Germano Granja, Clínica do Coração, Ouricuri, PE. Giovanni Saraiva, Imedi e Icordis, Recife, PE. Gleidson Junio Oliveira de Souza, Liga de Hipertensão Arterial UFG, Goiânia, GO. Gustavo Barros - MCOR / Recife-PE. Gustavo Guimarães Moreira de Castro, ITACORDIS e Universidade Iguaçu – UNIG, Itaboraí e Nova Iguaçu, RJ. Jadil Francisco Fusturath Júnior, Cardio Service, Porto Velho, RO. José Wladimir Tambelli Pires, Clínica de Cardiologia, Itapetininga/SP. João Evaristo de Oliveira Dantas, Cardiomed/Multimed, São Luís, MA. João Félix de Morais Filho, Clínica Angiocárdio, Natal / RN. João Francisco Martins Pacheco, Endocardio, Belém, PA. Jonathan Scapin Zagatti, Cardio Ritmo Diagnósticos, Jales, SP. José Joaquim Raposo, Serviço de Cardiologia da Santa Casa de Limeira, SP. José Roberto Moya, Biocardios, Cuiabá, MT. Josafá de Oliveira Costa, Clínica Vitta, Igarassu, PE. Josiedson Pontes de Farias, Cardio Diagnósticos, Caruaru, PE. Juan Carlos Yugar Toledo. Endocor, Rio Preto, SP. Lilian Mesquita, Ergo Med Setor De Cardiologia/Geriatria, RJ. Lola Helbingen Santos, Cardiodiagnósticos, Goiania, GO. Luam Vieira de Almeida Diógenes, Procardiaco, Teresina, PI. Luiz Kencis Júnior, Lapacor, São Paulo, SP. Marcelo Júlio de Oliveira, Clínica Cardiograficos, Ribeirão Preto, SP. Marco Antônio de M Alves, Escada Clinical Center, Escada, PE. Marco A. M. Gomes, Centro de Pesquisas Clínicas do Cesmac/Hospital do Coração de Alagoas. Marcos Alberto Pires Meira Júnior, Clincar João Pessoa PB. Maria Christina Cavalcanti Ballut, MEDCENTRO, Manaus, AM. Marcus Vinícius de Oliveira, Cardiodiagnósticos, Goiânia-GO. Maria Beatriz M. B. L. Rodrigues, Cardiovida, Porto Velho, RO. Mayara Cedrim Santos, Instituto UNICAP de Pesquisa Clínica, Recife, PE. Naiara Pedrassi Engracia Garcia Caluz, Centro de Medicina Avançada Dr Luiz Kencis, São Paulo, SP. Nelson Dinamarco, Ambulatório de Hipertensão Arterial, Colegiado de Medicina, Universidade Estadual Santa Cruz – UESC. Nildo Magalhães, SOBAM Jundiaí, SP. Paulo Roberto Pereira de Sant’Ana, MEDCOR Cardiologia, Nova Iguaçu- RJ. Rafael Nogueira de Macedo, Centro Cardiológico São Camilo, CE. Paulo Sérgio Lopes Soares, Universidade De Vassouras - Hospital Universitário de Vassouras, RJ. Ricardo Mesquita de Freitas, Cardiocenter, Barreiras, BA. Roberto de A. Dultra, Clinicor, Itabuna, Bahia, BA. Roberto Dischinger Miranda, Serviço de Cardiologia, Disciplina de Geriatria e Gerontologia, Escola Paulista de Medicina, Universidade Federal de São Paulo. Rodrigo Cunha de Sousa, Centro Integrado de Medicina Invasiva – CIMI, Uberaba, MG. Rogério Krakauer, Santa Casa de SP. Rogerio Ruiz, HD HomeDoctor, SP. Ruy Morando, Cincor - Centro Integrado do Coração, Americana, SP. Sérgio Augusto Vieira Simõe, Consultório Médico Integrado, Tássia Tâmara Silva Feitosa, Ok Doutor, Recife, PE. Tobias Barreto, SE. Sheyla Cristina Tonheiro Ferro da Silva, CLINSAUDE e CEMISE, Aracaju, SE. Vanderlei Magalhães da Silveira, Cardiologista, Faculdade de Medicina, Universidade de Passo Fundo. Vanildo Guimarães, Diagnóstico Cardíaco, Recife, PE. Vilma Helena Burlamaqui, Consultório de Cardiologia, Niterói, RJ. Vitor Bruno Teixeira de Holanda, Climile, Ananindeua, PA. Walmir de Vasconcelos Ratier Thomaz, Rio de Janeiro, RJ. Weimar Sebba Barroso, Liga de Hipertensão Arterial UFG, Goiânia, GO. Wenderson Tavares dos Santos, Hospital Biocor, Belo Horizonte, MG
